# Patterns of insecticide resistance in *Aedes aegypti* and *Aedes albopictus* in different cities across Cameroon

**DOI:** 10.1371/journal.pone.0361227

**Published:** 2026-10-28

**Authors:** Borel Djiappi-Tchamen, Carine Djapoum Theno, Stella Nana-Ndjangwo, Konstantinos Mavridis, Sofia Balaska, Abdou Talipouo, Steve Joko, Landre Djamouko-Djonkam, Roland Bamou, Audrey Marie Paul Mayi, Basile Kamgang, Parfait Awono-Ambéné, Gisele Aurelie Foko Dadji, John Vontas, Christophe Antonio-Nkondjio

**Affiliations:** 1 Institut de Recherche de Yaoundé (IRY), Organisation de Coordination pour la lutte Contre les Endémies en Afrique Centrale (OCEAC), Yaoundé, Cameroon; 2 Department of Animal Physiology and Biology, Faculty of Science, University of Yaoundé I, Yaoundé, Cameroon; 3 Institute of Molecular Biology and Biotechnology, Foundation for Research and Technology-Hellas, Heraklion, Greece; 4 Pesticide Science Laboratory, Department of Crop Science, Agricultural University of Athens, Athens, Greece; 5 Laboratory of Malaria and Vector Research, National Institute of Allergy and Infectious Diseases (NIAID), National Institutes of the Health (NIH), Rockville, Maryland, United States of America; 6 Department of Microbiology, Faculty of Science, University of Yaoundé I, Yaoundé, Cameroon; 7 Centre for Research in Infectious Diseases, Yaoundé, Cameroon; 8 Higher Teachers’ Training College of the University of Yaoundé I, Yaoundé, Cameroon; CEA, FRANCE

## Abstract

The increasing frequency of arboviral diseases’ outbreaks in Cameroon necessitates evidence-based vector control. However, the efficacy of chemical control interventions is seriously threatened by the rapid evolution of insecticide resistance in *Aedes* vector populations. This study investigated the insecticide resistance profiles of *Aedes aegypti* and *Aedes albopictus* across ecologically diverse cities in Cameroon. *Aedes* larvae were collected from April to November 2024 in eight major cities representing diverse ecological landscapes across Cameroon: Yaoundé (Congo-Guinean equatorial forest); Douala and Kribi (coastal areas); Nkongsamba, Bafoussam, Mbouda, and Maroua (highland areas); and Bertoua (sub-tropical/savanna transition zone). World Health Organization (WHO) insecticide susceptibility bioassays were conducted on F1 adults against pyrethroids (0.75% permethrin, 0.05% deltamethrin), carbamates (0.1% propoxur, 0.1% bendiocarb), and organophosphates (0.05% pirimiphos-methyl). Molecular mechanisms potentially driving insecticide resistance were characterized using multiplex TaqMan qPCR to genotype *kdr* mutations and RT-qPCR to quantify the expression of detoxification genes such as cytochrome P450s, Glutathione S-transferases, and Carboxylesterases. *Ae. aegypti* displayed widespread resistance to pyrethroids, with mortality as low as 17.4% (permethrin, Kribi). This resistance could possibly be attributed to distinct geographic distribution of target-site mutations, with F1534C ranging from absent in Bafoussam to 95% in Kribi and Bertoua (alongside high frequencies in Yaoundé and Douala), and V1016I peaking at 46.3% in Bertoua while remaining absent in Bafoussam and Maroua. Furthermore, double-mutant homozygous haplotypes (CC-II) reached up to 15%, alongside massive metabolic upregulation, notably a 184.8-fold increase in GSTD4 expression in Bafoussam. In contrast, *Ae albopictus* populations appeared generally more susceptible across most sites, though localized resistance to permethrin and deltamethrin emerged in Douala and Nkongsamba. Susceptibility to bendiocarb and pirimiphos-methyl was observed across all sites for both species. In contrast, most of these populations were found to be resistant to propoxur. The continued susceptibility of *Ae. albopictus* in most regions, contrasted with its emerging resistance in urban centers, underscores the need for species-specific and geographically tailored management interventions. Overall, these findings emphasize the urgent need to deploy targeted larviciding approaches in breeding sites and integrate routine resistance monitoring to mitigate the spread of arboviral vectors across Cameroon.

## Introduction

The growing expansion of arbovirus vectors driven by factors such as globalization, rapid urbanization, and climate change is now increasing the threat of vector borne diseases [1]. Such diseases include dengue, Yellow fever, and chikungunya; with dengue being the most widespread, across the world [2]. Up to 171,991 dengue cases were reported in the WHO African Region alone in 2023 [3]. Recent dengue outbreaks in Burkina Faso, Nigeria, and Senegal were responsible for over 700 deaths [4–7]. Countries in the central Africa region such as Democratic Republic of Congo and Chad also reported outbreaks but at a more limited scale [3,8–12].

In Cameroon, sporadic cases of dengue, yellow fever, and chikungunya are frequently reported in major urban centers [13–16]. This situation could be associated with the high abundance of competent vectors such as *Aedes aegypti* and *Aedes albopictus* [17–21]. The risk of arboviral disease outbreaks particularly dengue and yellow fever is estimated to be high in major cities due to the multiplication of suitable breeding habitats, poor waste management, water storage practices, abundance of vector, competent vector, and circulating virus [15,22,23]. Given the current absence of specific efficient treatments or vaccines for most of these arboviral diseases, vector control remains the primary prevention strategy.

In Cameroon, as in most part of Africa, insecticide-based interventions represent the primary strategy for controlling vector-borne diseases and have historically driven substantial reductions in disease transmission and vector population densities over the past two decades. In Cameroon, public health vector control is primarily directed toward malaria prevention through the mass distribution of long-lasting insecticidal nets (LLINs) treated with pyrethroids (such as permethrin and deltamethrin) between 2000 and 2023 [24]. While other vector control methods such as Indoor Residual Spraying (IRS) are not yet implemented in the disease control framework, recent findings reveal a shift in the biting behavior of *Aedes* populations [25,26], which exhibit endophilic tendencies during daytime and early evening hours. This behavior influences their exposure to the unregulated use of domestic aerosol sprays including carbamates and organophosphates and agricultural pesticides in urban areas.

However, the emergence of insecticide resistance poses a risk to the effectiveness and sustainability of the currently available insecticide based interventions. Recent studies in Cameroon indicated a rise in pyrethroid resistance in *Ae. aegypti*, linked to multiple point mutations (F1534C, V1016I, V410L) identified in the voltage-gated sodium channel (*Vgsc*) gene [18,27,28]. Mutations such as the V1016G or V1016I and F1534C have been reported to act by increasing the resistance status particularly when they are associated with S989P mutation [29]. Additional mechanisms including reduced cuticle penetration (cuticle thickening) have also been reported in *Aedes* species from Cameroon [30].

In addition to target-site mutations, over-expression of detoxification enzymes, mainly cytochrome P450 oxidases, esterases, and Glutathione-S transferases (GST), have been reported to induce resistance in *Ae. aegypti* populations originating from Douala and Yaoundé [18,27].

Studies conducted so far in the country reported the co-occurrence of both *Ae. albopictus* and *Ae. aegypti* with a predominance of the former species almost everywhere across south Cameroon [17,21]. Although they share similar habitats, they display different resistance profile. Mutation F1534C is present in low frequency in *Ae. albopictus*, contrary to *Ae. aegypti* in major Cameroonian cities [18,27]. In certain populations across Cameroon, resistance in *Ae. albopictus* has been linked primarily to the overexpression of *Cyp6* metabolic gene families [27,28].

The present study aims to assess the geographic distribution and patterns of insecticide resistance in *Ae.aegypti* and *Ae. albopictus* in main cities across Cameroon. The findings would support insecticides resistance management efforts in the fight of vector-borne diseases

## Materials and methods

### Study sites and mosquito sampling

Aedes immature stages were collected in eight cities (Yaoundé, Douala, Kribi, Nkongsamba, Bafoussam, Mbouda, Maroua, and Bertoua) across Cameroon, selected based on their distinct eco-epidemiological features and altitudinal variations. Because the larval surveys were conducted in public spaces and accessible private properties (residential areas and tire-repair shops/garages), formal written permits were not required by local authorities. However, prior oral informed consent was obtained from household heads or garage owners before inspecting potential larval habitats on their property. No private property was entered without explicit oral permission from the occupants. These sites represent a broad range of ecological zones including equatorial forests, coastal areas, highlands, and savannah landscapes.

Entomological collections were conducted from April to November 2024 corresponding to short and long rainy seasons. The descriptive features of each site are presented in Table 1. During each entomological survey, all water containers including discarded plastics containers, metallic containers, and used tires were inspected for *Aedes* immature stages presence. Larvae and pupae were preliminarily identified in the field as *Aedes* based on their characteristic morphological and behavioral features (such as the short, stout siphon and distinctive hanging/wriggling posture compared to *Culex*), and later confirmed in the insectary upon emergence. Positive containers were sampled using the deeping method, and the collected immature stages (larvae and pupae) were pooled (per city) and transported to the insectary of the Malaria Research Laboratory at Organisation de Coordination pour la lutte Contre les Endémies en Afrique Centrale (OCEAC).

**Table 1:** Description of study sites.

City	Ecological zone	Altitude	GPS Coordinates
Yaoundé	Congo-Guinean equatorial forest	[605-1209]	3° 52′ North; 11° 31′ East
Douala	Coastal area	[−27:317]	4° 03′ North; 9° 42′ East
Nkongsamba	Highland area	[520-1469]	4° 57′ North; 9° 56′ East
Kribi	Coastal area	[−1:1084]	2° 57′ North; 9° 55′ East
Bafoussam	Highland area	[987:1590]	5° 28′ North; 10° 25′ East
Mbouda	Highland area	[1071:1496]	5° 37′ North; 10° 15′ East
Bertoua	Sub-tropical	[603-779]	4° 34′ 30″ North; 3° 41′ 04″ East
Maroua	Highland area	[324:933]	10° 35′ North; 14° 19′ East

### *Aedes* mosquito rearing

Rearing of immature stages to adult specimens were performed under standard laboratory conditions (27–28 ◦C temperature; 70–80% relative humidity). Pupae were collected daily and transferred in cages until adult emergence. Adult mosquitoes were provided with 10% glucose solution. Morphological identification of *Aedes* species from each study site was done using a stereomicroscope with morphological keys [31].

### Insecticide susceptibility tests

Each specie was fed on chicken blood for egg-laying, and F1-generation adult females were used for insecticide susceptibility testing. A first subset of 50–60 non-blood fed mosquito females aged 3–5 days, unexposed to insecticides, were preserved in RNA later (SIGMA Aldrich, Saint Louis, MO, USA) for downstream molecular analyses of insecticide resistance. Bioassays were performed using standard WHO protocols [32] with the following insecticides classes: pyrethroids (0.75% permethrin, 0.05% deltamethrin), carbamates (0.1% propoxur, 0.1% bendiocarb), and organophosphates (0.05% pirimiphos-methyl). Batches of 20–25 females (four tubes) were exposed to insecticide-impregnated papers for 1 hour. For each test, two batches (two tubes) of 20–25 females were exposed to untreated papers as control. After bioassays, a subset of mosquito samples from each category (survivors, dead, control) was preserved in RNA later and shipped to IMBB-FORTH (Institute of Molecular Biology and Biotechnology, Foundation for Research and Technology-Hellas) for molecular analyses. The remaining mosquitoes were preserved in alcohol 70%.

### DNA extraction from individual mosquitoes

For species identification and target-site mutation genotyping, genomic DNA (gDNA) was extracted from individual mosquito legs for a total of N = 300 *Ae. aegyp*ti and N = 181 *Ae. albopictus*. Legs were homogenized in 200 μl of DNAzol Reagent (Molecular Research Center, Inc., purchased from Thermo) using a battery-powered tissue grinder and a plastic pestle in a 1.5 ml microcentrifuge tube. DNA extraction followed the manufacturer’s protocol with specific modifications: DNA precipitation was performed using 50 μl of absolute ethanol, and the precipitated DNA was pelleted by centrifugation at 10,000 × g for 12 minutes at 4°C. The pellet was then washed twice with 500 μl of 75% ethanol, followed by centrifugation at 10,000 × g for 5 minutes. After air-drying for 15 minutes on a heat block set to 50°C, the DNA was resuspended in 10 μl of DNA/DNase/RNase-free water and stored at −20°C.

### Total RNA extraction from mosquito pools

Total RNA from survivors of insecticide bioassays was extracted from pooled mosquito specimens (N = 10 per pool; 3 pools from each population) using the Trizol Reagent protocol, according to the manufacturer’s instructions. The quantity and purity of total RNA were assessed using a Nanodrop. The quality of total RNA was assessed by 1.0% weight/volume agarose gel electrophoresis.

### Molecular species identification

Molecular identification of both *Ae. aegypti* and *Ae. albopictus* species was performed using the TaqMan assay of Kothera et al.[33].

### Genotyping of knockdown resistance mutations in *Aedes aegypti*

Custom-developed multiplex TaqMan qPCR genotyping assays were used to detect *kdr* mutations (F1534C, V1016G, V1016I, and S989P), following the methodology described in Mavridis et al [34],. Reactions were performed in the CFX Opus Real-Time PCR System (Bio-Rad) using a one-step RT-PCR mastermix supplied by FTD (Fast-track diagnostics, Luxembourg) in a total reaction volume of 10 μL. The thermal cycle parameters were: 50°C for 15 min, 95°C for 3 min, and 40 cycles of 95°C for 3 second, and 60°C for 30 sec, allowing a sample to result time of ~75 min. Samples were amplified in duplicates, and each run always included a non-template control. The presence of the L982W substitution in the *VGSC* gene was assessed using PCR followed by Sanger sequencing, following the protocol described by Kasai et al [35],. A modification was applied in the sequencing step, where the AaeSCF20 primer was used in place of AaeSCF9 for improved coverage of the target region.

### Expression analysis for detoxification genes linked to insecticide resistance in *Aedes aegypti*

Custom-developed multiplex TaqMan RT-qPCR assays were used to quantify the expression of eight detoxification genes (*CYP6BB2, CYP9J26, GSTD4, CCEae3A, CYP9J28, CYP9M6, CYP9J32,* and *GSTE2*) [36], with *RPL8* included for normalization in each assay, following the methodology described by Mavridis et al, [34]. The Real-Time PCR reaction conditions were identical to those described in the previous section.

### Genotyping of knockdown resistance mutations in *Aedes albopictus*

The voltage-gated sodium channel (*VGSC*) domain II was investigated for the presence of the V1016G mutation and domain III for mutations I1532T and F1534L/S/C *via* PCR and product sequencing. The PCR (KAPA Taq PCR Kit) for domain II was carried out in 25 μl containing 1.5 μl of mixed gDNA extracted individually from 5–8 *Ae. albopictus* samples of the same locality, 2.5 μl of 10Χ DNA polymerase buffer, 0.4 μM of each primer, 0.4 μM of dNTPs and 1.5 U of Taq polymerase. The PCR thermal conditions are described as follows: initial denaturation at 95°C for 5 min, 40 cycles Χ (denaturation at 94°C for 30 s, primer annealing at 55°C for 30 s, primer extension at 72°C for 1 min) and a final extension step at 72°C for 5 min. A small amount of the PCR products (5 μl) was electrophoresed on a 1% w/v agarose gel to verify the presence of the correct size amplicon (500 bp), and the remaining amount was purified using the Nucleospin PCR and Gel Clean-Up Kit (Macherey Nagel) and sequenced using the Sanger method (GENEWIZ, Leipzig, Germany). Sequences were analysed using the sequence alignment editor BioEdit 7.2.5. The PCR for domain III was carried out in 25μl containing 1.5μl of gDNA from *Ae. albopictus* individuals, 2.5 μl of 10 × DNA polymerase buffer, 2mM MgCl2, 0.3 μM of each primer, 0.4uM of dNTPs and 1.5 U of Taq polymerase. The thermal conditions of the PCR were: initial denaturation at 95°C for 5 min, 40 cycles × (denaturation at 94°C for 30sec, primer annealing at 57°C for 30sec, primer extension at 72°C for 1 min) and a final extension step at 72°C for 5 min. The products were electrophoresed on a 1% w/v agarose gel and the specific 740 bp band was gel extracted and purified using the Nucleospin PCR and Gel Clean-Up Kit (Macherey Nagel, Dueren, Germany) and sequenced using the Sanger method (GENEWIZ, Leipzig, Germany). Sequences were analyzed using the sequence alignment editor BioEdit 7.2.5. The list of primers used can be found in Balaska et al, [37].

### Data analysis

Adult mortality was evaluated 24h post insecticide exposure and the susceptibility status was determined according to WHO criteria: confirmed resistance if mortality <90%, possible resistance if mortality is between 90 and 98%, and susceptible if mortality >98% [32]. For *kdr* target-site mutation analysis, mutant allele frequency (% MAF) was determined for each mutation using the equation:


$$\begin{array}{c} \%\;MAF\;=\;\left(\frac{(\text{2}\;\times \;hom\;+\;het}{\text{2}N}\right)\;\times \;\text{1}\text{0}\text{0} \end{array}$$ (1)

where N represents the total number of mosquitoes analyzed in each case. Haplotype frequencie**s** were determined using the equation:


$$\begin{array}{c} \%\;Haplotype\;Frequency=\;(\frac{N\;of\;individuals\;bearing\;each\;haplotype}{total\;N\;of\;individuals}\backslash \times \;\text{1}\text{0}\text{0} \end{array}$$ (2)

For detoxification gene expression analysis, fold-change calculations, 95% confidence intervals (CIs), and statistical significance were assessed following the Pfaffl method [38].

## Results

### Insecticide susceptibility assays in *Aedes aegypti* and *Aedes albopictus* populations

Despite the difference in the number of assays per locality due to the low number of collected specimens, and availability of insecticides, distinct resistance patterns were observed in both of the species (Fig 1).

**Fig 1:**
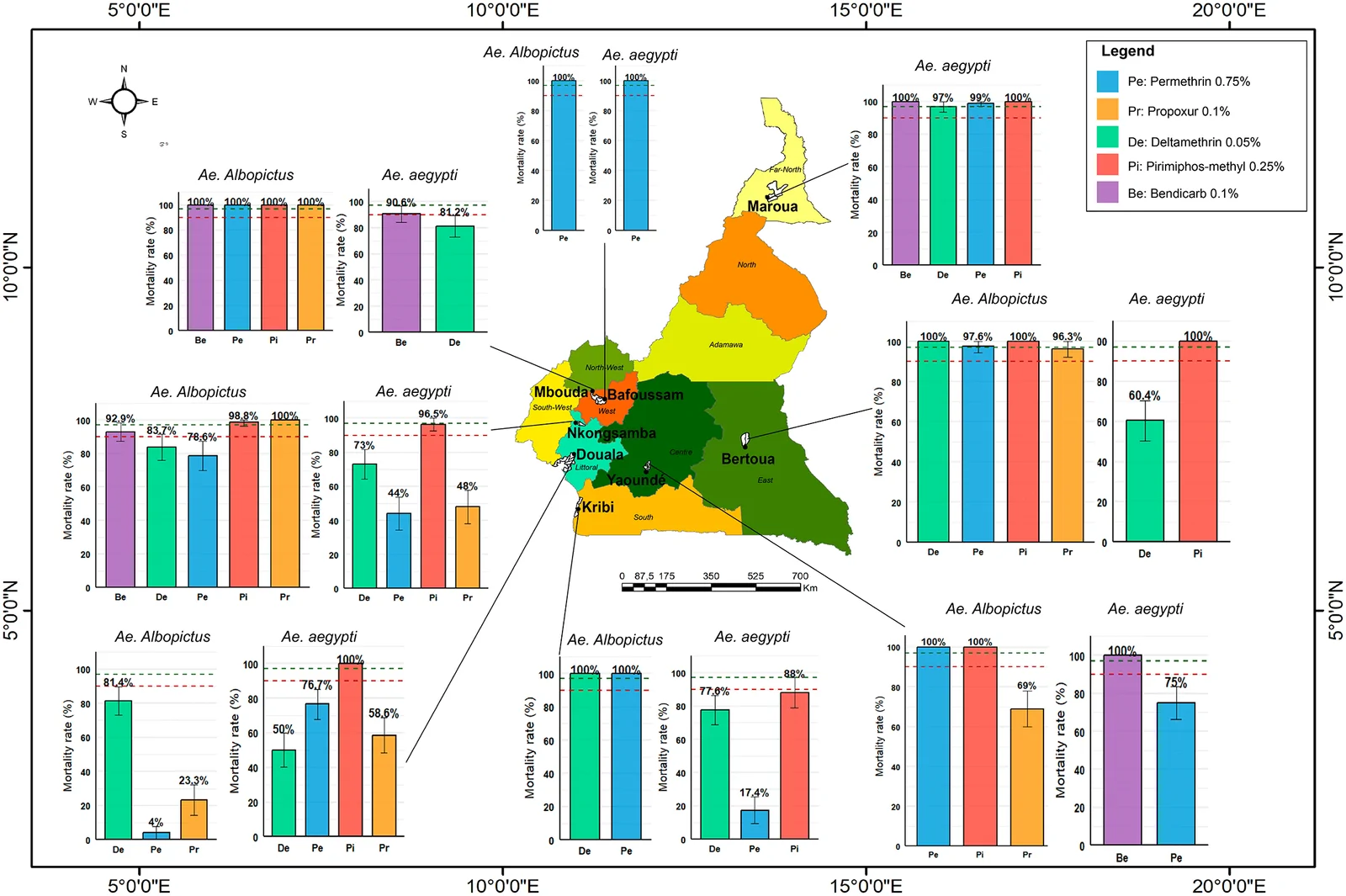
Mortality rates of *Aedes aegypti* and *Aedes albopictus* populations upon exposure to different insecticides; The dashed horizontal lines represent the WHO suspected resistance thresholds (90% and 97%).

*Aedes aegypti* populations from several locations, such as Bertoua, Bafoussam, Douala, Kribi, Maroua, Mbouda, Nkongsamba and Yaoundé displayed resistance to pyrethroids (deltamethrin 0.05%, permethrin 0.75%) with mortality rates varying from 17.4% against permethrin in Kribi, to 44% in Nkongsamba. Reduced susceptibility to the Carbamate Propoxur 0.1% was also recorded in Douala (58.6%) and Nkongsamba (48%), whereas suspected resistance to Bendiocarb 0.1% (mortality rates ≥90%) was recorded in almost all sites. Regarding the organophosphate Pirimiphos-methyl 0.25%, female mosquitoes from Kribi exhibited resistance (88%), while a suspected resistance (96.5%) was recorded in Nkongsamba; in contrast, susceptibility was detected in populations from Maroua, Douala, and Bertoua (100%).

Mortality rates of Aedes albopictus populations exposed to WHO diagnostic doses of insecticides indicates general susceptibility to the insecticides tested in the majority (5/8) of sites. However, resistance to permethrin 0.75% and Deltamethrin 0.05% were recorded in Douala (4% and 81.4%) and Nkongsamba (78.6% and 83.7%). Additionally, resistance to the Propoxur was detected in Douala (23.3%) and Yaoundé (69%).

### Target site mutations and haplotype analysis in *Aedes aegypti* populations

Analysis of target site mutations in eight Ae. aegypti (Table 2) populations revealed substantial variations in the frequency of the F1534C mutation, ranging from 1.3% in Maroua to 95% in Kribi and Bertoua. High frequencies were also observed in Yaoundé (91.3%), Douala (78.8%), and Nkongsamba (68.8%), whereas this mutation was absent in Bafoussam. The V1016I mutation was detected in multiple populations, with the highest frequency recorded in Bertoua (46.3%), followed by Yaoundé (32.5%), Nkongsamba (28.8%), Douala (25.0%), Kribi (23.8%), and Mbouda (13.8%). This mutation was not detected in both Bafoussam and Maroua. The S989P and L982W mutations were all not detected in any of the populations examined.

**Table 2:** Allelic frequencies of *kdr* mutations within the Voltage-Gated Sodium Channel gene in *Aedes aegypti* populations.

Cities	F1534C (A/B)	V1016G (A/B)	V1016I (A/B)	S989P (A/B)	L982W (A/B)
Yaoundé	**91.3%**(3/35) n = 80	**0.0%**(0/0) n = 80	**32.5%**(20/3) n = 80	**0.0%**(0/0) n = 80	**0.0%**(0/0) n = 32
Douala	**78.8%**(1/31) n = 80	**0.0%**(0/0) n = 80	**25.0%**(14/3) n = 80	**0.0%**(0/0) n = 80	**0.0%**(0/0) n = 38
Nkongsamba	**68.8%**(23/16) n = 80	**0.0%**(0/0) n = 80	**28.8%**(17/3) n = 80	**0.0%**(0/0) n = 80	**0.0%**(0/0) n = 36
Kribi	**95.0%**(0/38) n = 80	**0.0%**(0/0) n = 80	**23.8%**(15/2) n = 80	**0.0%**(0/0) n = 80	**0.0%**(0/0) n = 40
Maroua	**1.25%**(1/0) n = 80	**0.0%**(0/0) n = 80	**0.0%**(0/0) n = 80	**0.0%**(0/0) n = 80	**0.0%**(0/0) n = 10
Mbouda	**38.8%**(15/8) n = 80	**0.0%**(0/0) n = 80	**13.8%**(7/2) n = 80	**0.0%**(0/0) n = 80	**0.0%**(0/0) n = 44
Bertoua	**95.0%**(4/36) n = 80	**0.0%**(0/0) n = 80	**46.3%**(23/7) n = 80	**0.0%**(0/0) n = 80	**0.0%**(0/0) n = 24
Bafoussam	**0.0%**(0/0) n = 40	**0.0%**(0/0) n = 40	**0.0%**(0/0) n = 40	**0.0%**(0/0) n = 40	**0.0%**(0/0) n = 4

A/B = number of heterozygous/homozygous for the *kdr* mutation; n = number of alleles

Haplotype analysis of *VGSC* loci F1534C, V1016I, and S989P (Fig 2) revealed substantial variation across the study sites. In Bertoua, some mosquitoes had the following combination of haplotypes CC-II-SS, CC-VI-SS, FC-VI-SS, and FC-II-SS which accounted for 75% of the haplotypes. In Yaoundé, the percentage of mosquitoes concurrently bearing two mutations (CC-II-SS, CC-VI-SS, FC-VI-SS) was 55%. In Nkongsamba, 50% of mosquitoes had haplotypes combining F1534C and V1016I mutations (CC-II-SS, CC-VI-SS, FC-VI-SS). In Kribi, the frequency of mosquitoes with dual mutations was 42.5% (CC-II-SS and CC-VI-SS). In Douala, 25% of mosquitoes carried combined resistance haplotypes (CC-II-SS, CC-VI-SS, FC-VI-SS). In Mbouda, 22.5% of mosquitoes carried double-mutation haplotypes (CC-II-SS, CC-VI-SS, FC-VI-SS). While in Bafoussam only the fully susceptible haplotype VV-FF-SS was detected (100%).

**Fig 2:**
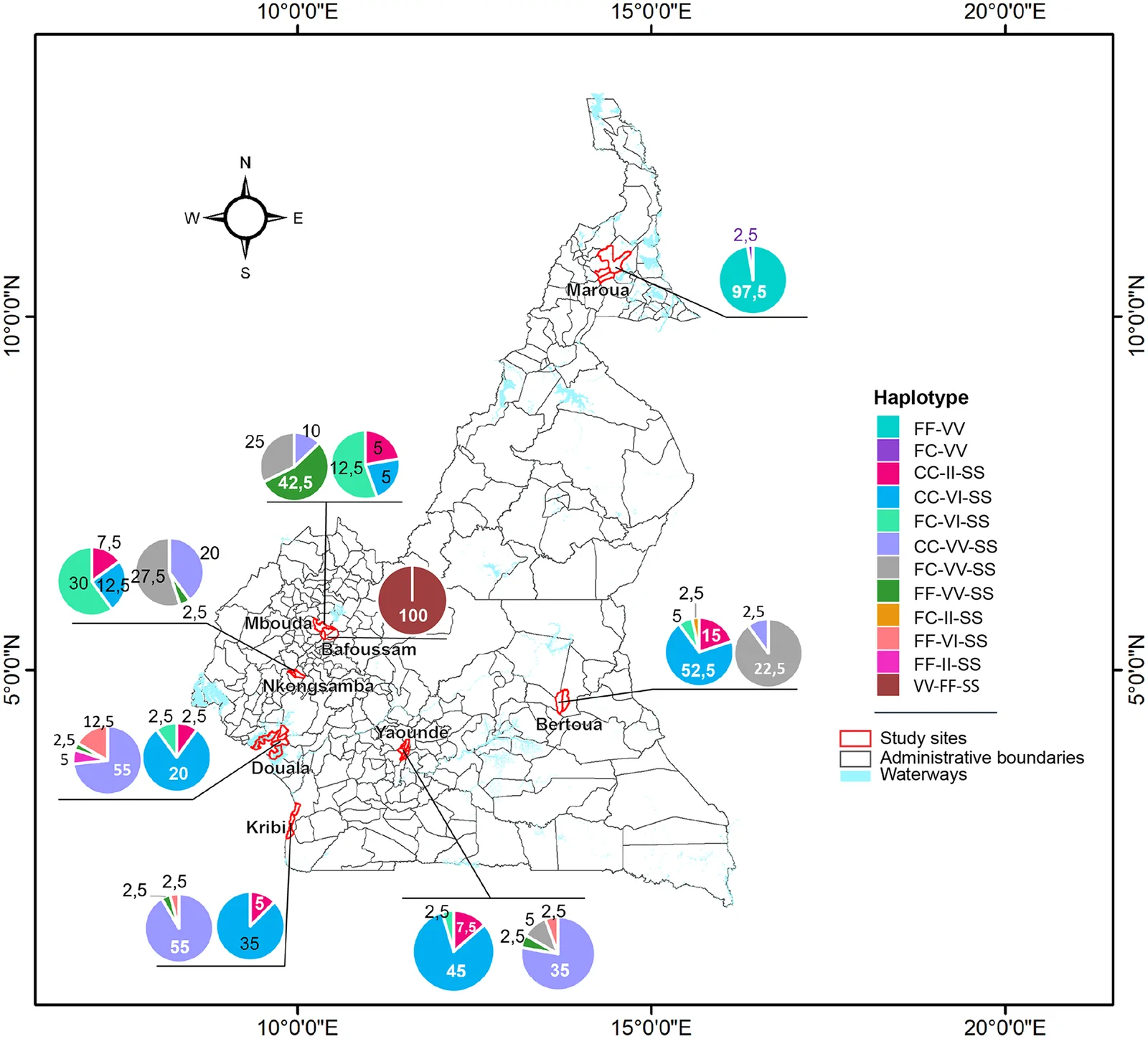
Haplotype frequencies in *Aedes aegypti* populations for *Vgsc* loci F1534C, V1016I and S989P in different cities of Cameroon.

### Target site mutations in *Aedes albopictus* populations

*Aedes albopictus* populations from the different sites were screened for the following *kdr* mutations V1016G, I1532T, F1534C, F1534L, and F1534S (Table 3). Out of the 5 loci screened, F1534S was detected in Douala at very low frequency (1.35%) and F1534C was detected in Nkongsamba at a frequency of 6.82%.

**Table 3:** Allelic frequencies of *kdr* mutations within the Voltage-Gated Sodium Channel gene in *Aedes albopictus* populations.

Cities	V1016G (A/B)	I1532T (A/B)	F1534C (A/B)	F1534L (A/B)	F1534S (A/B)
Yaoundé	**0.0%**(0/36) n = 36	**0.0%**(0/36) n = 36	**0.0%**(0/36) n = 36	**0.0%**(0/36) n = 36	**0.0%**(0/36) n = 36
Douala	**0.0%**(0/74) n = 74	**0.0%**(0/74) n = 74	**0.0%**(0/74) n = 74	**0.0%**(0/74) n = 74	**1.35%**(0/74) n = 74
Nkongsamba	**0.0%**(0/88) n = 88	**0.0%**(0/88) n = 88	**6.82%**(0/88) n = 88	**0.0%**(0/88) n = 88	**0.0%**(0/88) n = 88
Kribi	**0.0%**(0/52) n = 52	**0.0%**(0/52) n = 52	**0.0%**(0/52) n = 52	**0.0%**(0/52) n = 52	**0.0%**(0/52) n = 52
Mbouda	**0.0%**(0/40) n = 40	**0.0%**(0/40) n = 40	**0.0%**(0/40) n = 40	**0.0%**(0/40) n = 40	**0.0%**(0/40) n = 40
Bertoua	**0.0%**(0/34) n = 34	**0.0%**(0/34) n = 34	**0.0%**(0/34) n = 34	**0.0%**(0/34) n = 34	**0.0%**(0/34) n = 34
Bafoussam	**0.0%**(0/38) n = 38	**0.0%**(0/38) n = 38	**0.0%**(0/38) n = 38	**0.0%**(0/38) n = 38	**0.0%**(0/38) n = 38

A/B= number of heterozygous/homozygous; n= number of alleles

### Expression of detoxification genes in *Aedes aegypti* populations

The expression levels of seven major detoxification genes involved in insecticide resistance in Ae. aegypti (CYP6BB2, CYP9J26, CYP9J28, CYP9J32, CYP9M6, GSTD4, GSTE2, and CCEae3A) were analyzed using multiplex TaqMan RT-qPCR assays using survivors of the bioassays (Table 4). Two P450 genes CYP9J28 and CYP9M6 were detected significantly overexpressed (P < 0.001); CYP9J28 was notably upregulated in Bafoussam (18.5-fold) and Mbouda (2.82-fold), while CYP9M6 showed overexpression across multiple populations, including Bafoussam (2.73-fold), Kribi (2.84-fold), Mbouda (2.60-fold), and Nkongsamba (2.06-fold). In contrast, CYP6BB2, CYP9J26, CCEae3A, CYP9J32, and GSTE2 did not exhibit statistically significant overexpression values in any of the populations examined. GSTD4 was significantly upregulated in all analyzed populations with expression levels ranging from approximately 7.5-fold in Nkongsamba to a remarkable 184.8-fold in Bafoussam.

**Table 4:** Expression analysis of detoxification genes in *Aedes aegypti* mosquito populations compared to susceptible mosquito strain.

City	*CYP6BB2*	*CYP9J26*	*GSTD4*	*CCEae3A*	*CYP9J28*	*CYP9M6*	*CYP9J32*	*GSTE2*
Yaoundé	0.17 (0.07 - 0.45) P = 0.012	0.74 (0.58 - 0.96) P < 0.001	12.2* (5.22 - 40.3) P < 0.001	0.44 (0.31 - 0.78) P < 0.001	1.09 (0.63 - 1.96) P = 0.732	1.49 (0.95 - 2.09) P = 0.156	0.14 (0.07 - 0.25) P < 0.001	1.36 = 6 (0.45 - 4.68) P = 0.761
Bafoussam	0.38 (0.10 - 1.21) P = 0.18	0.85 (0.67 - 1.08) P = 0.287	184.8* (25.8 - 932.1) P < 0.001	0.59 (0.36 - 1.19) P = 0.107	18.5* (13.3 - 25.6) P < 0.001	2.73* (2.38 - 3.24) P < 0.001	1.14 (0.51 - 2.18) P = 0.722	1.94 (0.62 - 7.24) P = 0.400
Douala	0.03 (0.018 - 0.06) P = 0.045	0.28 (0.20 - 0.41) P < 0.001	12.4* (3.52 - 40.3) P < 0.001	0.45 (0.29 - 0.80) P = 0.032	1.19 (0.67 - 1.88) P = 0.491	1.06 (0.81 - 1.42) P = 0.696	0.09 (0.04 - 0.25) P < 0.001	1.04 (0.68 - 1.48) P = 0.769
Kribi	0.45 (0.09 - 1.64) P = 0.38	0.92 (0.54 - 1.34) P = 0.718	18.9* (5.71 - 91.4) P = 0.032	0.59 (0.37 - 1.18) P = 0.111	1.14 (0.71 - 2.09) P = 0.622	2.84* (1.99 - 3.77) P = 0.035	0.31 (0.15 - 0.62) P = 0.045	1.15 (0.51 - 2.48) P = 0.625
Mbouda	0.23 (0.10 - 0.53) P < 0.001	0.93 (0.68 - 1.22) P = 0.645	12.2* (2.22 - 191.9) P < 0.001	0.46 (0.34 - 0.75) P < 0.001	2.82* (2.07 - 3.61) P < 0.001	2.60* (1.33 - 6.00) P = 0.032	0.56 (0.36 - 0.81) P < 0.001	1.35 (0.66 - 2.59) P = 0.395
Nkongsamba	0.16 (0.05 - 0.39) P < 0.001	0.50 (0.29 - 0.90) P = 0.029	7.46* (1.92 - 66.5) P = 0.033	0.36 (0.24 - 0.71) P < 0.001	1.93 (0.81 - 7.78) P = 0.471	2.06* (1.38 - 3.33) P < 0.001	0.20 (0.11 - 0.33) P = 0.039	0.98 (0.48 - 1.86) P = 0.889

* Bold letters indicate statistically significant overexpression at 95% Cis.

We didn’t investigate the expression of detoxification enzymes in *Ae. albopictus* populations.

## Discussion

Our findings regarding widespread pyrethroid resistance levels and high frequencies of target-site mutations (*F1534C* and *V1016I*) in *Ae. aegypti* align with recent baseline surveillance in major urban centers such as Yaoundé, Douala, and Bertoua [39]. Furthermore, while Keumeni et al. [39] demonstrated the initial involvement of *kdr* mutations across these cities, the present study expands upon this framework by incorporating a broader national scale across diverse ecological zones, detailed multi-locus haplotype combinations, and quantitative expression profiling of key detoxification genes. The resistant observed in *Ae. aegypti* may be driven by its high adaptive capacity to survive environmental stressors [21,40,41]. Furthermore, rapid urbanization and poor sanitation in many urban areas create prolific breeding sites [42], increasing human-vector contact driving frequent insecticide use for mosquito control, then intensifying selection pressure [43].

Despite their high population densities in Cameroon [22], *Ae. albopictus* consistently displays lower resistance to insecticides than sympatric *Ae. aegypti* [44]. This divergence may stem from behavior differences: *Ae. aegypti* is highly endophilic and anthropophilic, which exposes it to domestic aerosols and treated materials, whereas the peri-domestic, vegetated habitat of *Ae. albopictus* limit consistent chemical exposure [45,46]. Consequently, the relationship between these behavioral differences and differential insecticide exposure warrants further investigations. Nevertheless, heterogeneous resistance pattern emerged; Douala populations showed high resistance to permethrin and propoxur, whereas Nkongsamba and Yaoundé populations exhibited higher mortality rates to the same compounds. This localized resistance is likely driven by intensive pesticide use in urban and peri-urban farming, as the chemical families used in agricultural pest control overlap with those used in public health, creating a strong potential selection pressure as previously reported in Cameroon [47].

Pyrethoid resistance correlated with high frequencies of *VGSC* mutations, specifically F1534C and V1016I, aligning with previous reports in Douala and Yaoundé [18,39,48]. The F1534C mutation was highly frequent in Kribi (95%), Bertoua (95%), Yaoundé (91.3%) and Douala (78.8%). Meanwhile, the V1016I mutation, was detected in Bertoua (46.3%), Yaoundé (32.5%), Nkongsamba (28.8%), and Douala (25%).

The co-occurrence of multiple mutations on the same haplotype was identified in the studied populations. This finding implies a potential synergistic effect, that could lead high pyrethroid resistance levels. This mechanism is underscored by a recent study in Florida where, resistance intensity to pyrethroids (permethrin and deltamethrin) and mosquito survival increased proportionally with the number of co-occuring VGSC mutations, peaking in double-homozygous (II/CC) genotypes [48]. According to these authors, individuals carrying the V1016I and F1534C alleles displayed highest insecticide resistance rate compared to those with lower or reduce number of mutations. Additionally, laboratory study from Harris County in Texas analyzing the probability of survival for individual mutation sites, demonstrated that the CC genotype’s survival was similar to the FC heterozygote, while the II genotype had greater survival than the non-differentiating VI and VV genotypes. Overall, the double homozygous resistant genotype (II/CC) achieved the highest probability of survival [49]. If this situation is generalized, it will be critical for addressing arbovirus disease control in Cameroon, particularly concerning the wide presence of resistant genotypes and the nationwide emergence of diseases.

The widespread distribution and increasing frequency of these multi-locus mutation patterns also underscore the urgent need to integrate full haplotype genotyping rather than single-mutation screening into resistance monitoring frameworks. Without this level of resolution, vector control programs and public health interventions which currently lack dedicated *Aedes*-specific strategies and often rely on broad chemical tools or malaria-focused assets risk to underestimate the underlying molecular complexity, leading to operational failures in managing arbovirus vectors, particularly in Bertoua and Yaoundé where combined genotypes are highly frequent. Compared to our previous study [18], the proportion of double mutant haplotypes has increased in major urban centers such as in Bertoua (75%) and Yaoundé (55%). This shift may reflect geographically variable selection pressures driven by excalating insecticide use. Such concurrent presence of both homozygous and heterozygous mutant alleles in resistant *Ae. aegypti* populations mirrors trends across West Africa (Benin, Niger, and Burkina-Faso) and the Americas [48,50–52].

While *kdr* mutations are the primary molecular markers associated with pyrethroid resistance in *Ae. aegypti* in this study, they were significantly less prevalent in *Ae. albopictus*. Out of five known *kdr* mutations screened, we detected only F1534S and F1534C at low frequencies in Douala (1.35%) and Nkongsamba (6.82%), suggesting target-site mutations are the sole or dominant drivers of the observed resistance in Cameroonian *Ae. albopictus*. Similar trends were also reported in Central African Republic, Brazil, Costa Rica, and China [53–56]. However, characterizing the metabolic resistance profile of *Ae. albopictus* was beyond the scope of this study.

Analysis of detoxification enzymes in Ae. aegypti populations revealed a broader geographical distribution and expression profile of CYP9J28 and CYP9M6 than previously reported [39]. Upregulation of these cytochrome P450 families drives metabolic resistance by accelerating pyrethroid degradation [57,58]. This metabolic overexpression in synergy with target-site mutations, provide a robust correlation for the reduce mortality observed across major urban centers. Consequently, the widespread overexpression of *CYP9J28* and *CYP9M6* against type I and II pyrethroids underscores their critical role in driving metabolic resistance across Cameroon, then reinforcing their value as key molecular markers for resistance surveillance [18,28,39].

The CYP9J32 enzymes previously reported to be moderately overexpressed in both Yaoundé and Douala together with CYP6BB2, CYP9J26, CCEae3A, and GSTE2, all of which are known to play key roles in pyrethroid and organophosphate resistance [59], were not significantly overexpressed in any of our populations. This decline may stem from Cameroon’s current insecticide rotation strategy (including permethrin, deltamethrin, PBO+ permethrin/ deltamethrin, and Chlorfenapyr) which may not exert the same selective pressure required to maintain high expression levels of these detoxification genes. Consequently, this shifting chemical exposure can influence gene flow; migration and interbreeding with susceptible populations may dilute resistance-associated alleles [60]. Indeed, in the absence of sustained selection, random genetic drift could similarly reduce the frequency of these previously advantageous alleles [61].

GSTD4 was significantly upregulated in all analyzed populations, with expression levels ranging from approximately 7.5-fold in Nkongsamba to a remarkable 184.8-fold in Bafoussam. The GSTD4, alongside GSTE2 and GSTS1 genes were previously described to detoxify pyrethroids by catalyzing the conjugation of a reduced glutathione to the pyrethroid molecules, making them less toxic and easier to excrete [35,62,63].

The Bafoussam population provides a notable exception to the observed resistance patterns. Despite being fully susceptible to pyrethroids in our bioassays, this population exhibited the highest observed levels of *GSTD4* and *CYP9J28* overexpression. This discrepancy suggests that transcriptional upregulation of detoxification genes is not always sufficient to confer survival against WHO diagnostic doses of insecticides. It is possible that in Bafoussam, these genes are being up-regulated as a ‘pre-adaptive’ or stress-response mechanism due to environmental chemical exposure, but that the expression levels remain below the threshold required to confer phenotypic resistance. Alternatively, there may be post-transcriptional factors or differences in enzyme activity levels that limit the functional detoxification capacity of these populations. This finding reinforces the necessity of using phenotypic data as the primary metric for resistance surveillance, with molecular tools serving as indicators of underlying selective pressures rather than definitive predictors of resistance status. However, further investigations such as functional validation are required to fully untangle the specific role of GSTD4 in Cameroonian *Ae. aegypti.*

Overall, these findings demonstrate notable differences in the insecticide resistance pattern in both *Ae. aegypti* and *Ae. albopictus*. The study also underscores the importance to investigate different factors influencing insecticide susceptibilities across different ecological settings in Cameroon.

## Conclusion

Our results highlight a broad pattern of pyrethroid resistance affecting both *Aedes aegypti* and *Aedes albopictus* populations in Cameroon, with certain localized variations in resistance profile between the two species. *Ae. aegypti* populations across multiple urban centers exhibit both widespread target-site *kdr* mutations (F1534C, V1016I) and overexpression of key detoxification genes (CYP9J28, CYP9M6, GSTD4), reflecting a multifactorial resistance profile that threatens the efficacy of pyrethroid-based interventions. In contrast, *Ae. albopictus* shows a much lower frequency of *kdr* (F1534S, F1534C) mutations, indicating that metabolic detoxification are likely the primary drivers of resistance. The resistance patterns recorded during the present study, underscore the urgent need for species-specific approach for arboviral diseases control.

## Abreviations:

Ae: Aedes
VGSC: Voltage gated sodium channel
